# Decreased plasma cartilage acidic protein 1 in COVID‐19

**DOI:** 10.14814/phy2.15814

**Published:** 2023-09-04

**Authors:** Mats W. Johansson, Joseph Balnis, Laura K. Muehlbauer, Yury V. Bukhman, Matthew S. Stefely, Katherine A. Overmyer, Rachel Vancavage, Anupama Tiwari, Anish Raj Adhikari, Paul J. Feustel, Bradford S. Schwartz, Joshua J. Coon, Ron Stewart, Ariel Jaitovich, Deane F. Mosher

**Affiliations:** ^1^ Morgridge Institute for Research Madison Wisconsin USA; ^2^ Department of Biomolecular Chemistry University of Wisconsin‐Madison Madison Wisconsin USA; ^3^ Department of Medicine University of Wisconsin‐Madison Madison Wisconsin USA; ^4^ Division of Pulmonary and Critical Care Medicine Albany Medical Center Albany New York USA; ^5^ Department of Molecular and Cellular Physiology Albany Medical College Albany New York USA; ^6^ National Center for Quantitative Biology of Complex Systems Madison Wisconsin USA; ^7^ Department of Chemistry University of Wisconsin‐Madison Madison Wisconsin USA; ^8^ Department of Neuroscience and Experimental Therapeutics Albany Medical College Albany New York USA; ^9^ Present address: Division of Allergy, Pulmonary and Critical Care Medicine, Department of Medicine University of Wisconsin Madison Wisconsin USA

**Keywords:** CFP/properdin, COVID‐19, CRTAC1, Type 2 alveolar epithelial cells

## Abstract

Cartilage acidic protein‐1 (CRTAC1) is produced by several cell types, including Type 2 alveolar epithelial (T2AE) cells that are targeted by SARS‐CoV2. Plasma CRTAC1 is known based on proteomic surveys to be low in patients with severe COVID‐19. Using an ELISA, we found that patients treated for COVID‐19 in an ICU almost uniformly had plasma concentrations of CRTAC1 below those of healthy controls. Magnitude of decrease in CRTAC1 distinguished COVID‐19 from other causes of acute respiratory decompensation and correlated with established metrics of COVID‐19 severity. CRTAC1 concentrations below those of controls were found in some patients a year after hospitalization with COVID‐19, long COVID after less severe COVID‐19, or chronic obstructive pulmonary disease. Decreases in CRTAC1 in severe COVID‐19 correlated (*r* = 0.37, *p* = 0.0001) with decreases in CFP (properdin), which interacts with CRTAC1. Thus, decreases of CRTAC1 associated with severe COVID‐19 may result from loss of production by T2AE cells or co‐depletion with CFP. Determination of significance of and reasons behind decreased CRTAC1 concentration in a subset of patients with long COVID will require analysis of roles of preexisting lung disease, impact of prior acute COVID‐19, age, and other confounding variables in a larger number of patients.

## Graphical abstract

A double‐site ELISA measured deviations in the concentration of CRTAC1 from the normal range (shaded) in patients with acute and long COVID‐19. Decreases in CRTAC1 correlated with decreases in CFP, to which CRTAC1 bound in an interaction ELISA.

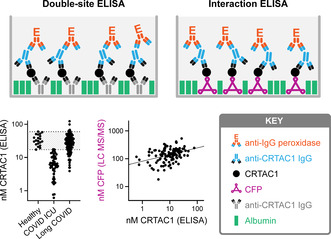

## 1 INTRODUCTION

In a mass spectrometric study conducted early in the COVID‐19 pandemic, we found decreases in plasma cartilage acidic protein 1 (CRTAC1) as measured by label‐free quantification (IBAQ) in a cohort of patients with COVID‐19 and deteriorating respiratory status (Overmyer et al., 2021). Similarly, CRTAC1 was among the circulating proteins reported by Byeon et al. (2022) and Shen et al. (2020) to be decreased in severe COVID‐19 in studies that used tandem mass tagging to compare subjects with non‐severe and severe disease. Although CRTAC1 is an understudied protein of obscure function, several observations suggest specific ties between CRTAC1 and COVID‐19. The gene for human CRTAC1 overlaps head‐to‐tail with the gene for GOLGA7B that is required for palmitoylation of SARS‐CoV2 spike protein and production of infectious virus (Wu et al., 2021). Among cell types producing CRTAC1 are Type 2 alveolar epithelial (T2AE) cells (Mayr et al., 2021), which are susceptible to infection by SARS‐CoV2 (Huang et al., 2020; Katsura et al., 2020). Expression of CRTAC1 in cultured T2AE cells increases upon treatment with dexamethasone (Ballard et al., 2010). CRTAC1 is decreased in plasma and bronchoalveolar lavage (BAL) of patients with idiopathic pulmonary fibrosis, a finding that has been attributed to loss of expression of CRTAC1 by de‐differentiated T2AE cells (Mayr et al., 2021). Finally, in a global screen of proteins interacting with a CRTAC1 bait in cell lysate, CFP (properdin of the alternate complement pathway) was one of the hits (Huttlin et al., 2021). Circulating CFP is decreased as part of the intense activation of the alternate pathway that accompanies severe COVID‐19 (Boussier et al., 2022; Siggins et al., 2023).

We now report molar concentrations of CRTAC1 as determined by an enzyme‐linked immunoassay (ELISA) in healthy normal controls and patients hospitalized with respiratory failure due to severe COVID‐19 or other causes, recovered from COVID‐19, or with chronic pulmonary disease (COPD). We found that concentrations in hospitalized patients with COVID‐19 fell to as low as 2% of the mean normal level and the magnitude of decrease correlates with severity indices. CRTAC1 correlated with mass spectrometric quantification of 173 other plasma proteins. The highest direct correlation was with CFP. We demonstrate that soluble recombinant CRTAC1 interacts with insolubilized recombinant CFP and propose that decreases of CRTAC1 associated with severe COVID‐19 result from increased turnover due to activation of the alternate complement pathway as well as to loss of production of CRTAC1 by dying or de‐differentiated T2AE cells. Finally, we found low plasma CRTAC1 in some patients a year after hospitalization with severe COVID‐19 or long COVID after COVID‐19 not requiring hospitalization. Further studies are needed to understand the reasons behind and significance of low CRTAC1 concentrations in long COVID.

## 2 MATERIALS AND METHODS

### 2.1 Samples

Plasma was from PPP vacutainer tubes that contained dried EDTA to avoid dilution of proteins with liquid anticoagulant. Samples of the 128 patients from our original study (Overmyer et al., 2021), 102 with COVID‐19 and 26 with respiratory deterioration from other causes, had been frozen at −80°C in April and May 2020. These were thawed, and 50‐μl aliquots were made, refrozen, shipped on dry ice, and kept at −80°C until the time of testing. Aliquots of plasma were prepared for five additional groups and handled in a similar manner. Twenty individuals who had no history of respiratory disease and were fully vaccinated against COVID‐19 were recruited in October 2021 and August 2022 as normal controls. Fifty‐five COPD patients recruited between May 2019 and May 2021 to an unpublished ongoing study of disease progression were studied as representative of patients at risk in the pandemic. None had a history of COVID‐19 or were undergoing an acute exacerbation or infection at the time of clinic visit. Five previously unstudied hospitalized patients with COVID‐19 were studied at 3‐day intervals in January 2021 to assess stability of CRTAC1 concentration. Participants from the original cohort (Overmyer et al., 2021) who survived COVID‐19 hospitalization were recontacted 1 year after discharge, and 16, corresponding to 30% of surviving individuals, consented to a second office visit in April and May 2021 for clinical evaluation and provided a new blood sample for analysis (Balnis et al., 2022). Finally, CRTAC1 concentration was determined in patients with long COVID recruited as part of study with a target enrollment of 250 and the goal of understanding the biological underpinnings of this condition. These patients were self‐referred to a dedicated post COVID‐19 clinic at Albany Medical Center with variable combinations of physical and cognitive symptoms at least 4 weeks after contracting COVID‐19 not requiring hospitalization as documented by PCR and/or antigen test. We studied the first 127 patients, who enrolled between February and June 2021 and between September and November 2022. A subset (*n* = 16) of these 127 long COVID patients had COPD comorbidity. We analyzed relationships of CRTAC1 concentration to interim aggregated patient metadata of the 102 patients enrolled before July 2021. Demographic characteristics of the study subjects are described in Table 1.

**TABLE 1:** Characteristics of study subjects.

Group	Total	Sex %male/female	Age mean (IQR)	Ethnicity
Healthy	20	45/55	48 (42–55)	65% White, 5% Black 25% Asian, 5% Hispanic
COPD	55	42/58	65 (59–71)	86% White, 14% Black
Severe non‐COVID	26	50/50	64 (53–77)	81% White, 15% Black 4% Hispanic
Severe COVID	102	63/37	61 (50–74)	47% White, 11% Black 14% Asian, 22% Hispanic, 6% Unknown
Severe COVID trajectory	5	20/80	58 (54–58)	80% White, 20% Black
Severe COVID at 1 year	16	50/50	53 (46–59)	38% White, 19% Black 19% Asian, 25% Hispanic
Long COVID	127	27/73	50 (41–58)	87% White, 6% Black 4% Asian, 1% Hispanic, 2% Unknown

Abbreviation: IQR, interquartile range.

### 2.2 ELISA

A double‐site ELISA was developed with reagents purchased from the R&D branch of BioTechne, Minneapolis, MN. The standard was recombinant human CRTAC1 lacking the signal peptide (residues 28‐661, catalog No. 5234‐CR‐050) produced as a secreted protein by NS0 mouse myeloma cells. This was supplied carrier‐free as a lyophilized powder and had no tendency to aggregate after being brought into solution as assessed by lack of absorbance at 320 nm. The absorbance at 280 nm matched the stated amount of protein in the vial as calculated assuming that a 1 mg/mL solution has an absorbance of 0.81 based on amino acid composition (https://web.expasy.org/cgi‐bin/protparam/protparam).

Wells of 96‐well EIA/RIA flat bottom high‐protein binding capacity polystyrene microtiter plates (Corning, Corning, NY) were coated overnight at 4°C with 50 ng affinity‐purified sheep polyclonal anti‐human CRTAC1 immunoglobulin (Ig) G (catalog No. AF5234), that is, with 50 μl of a 1 μg/ml solution in Tris‐buffered saline, pH 7.4 (TBS). Subsequent steps were performed at room temperature. Non‐adsorbed immune IgG was decanted, non‐occupied protein binding sites were blocked for 1 h with 200 μl 1% bovine serum albumin (BSA) in TBS‐0.05% Tween 20 (TBST), and wells were washed three times with TBST. Wells were then incubated for 1 h with 50 μl 0.2–200 ng/ml (0.0029–2.9 nM) CRTAC1 or unknown plasma diluted 1/30, 1/100, 1/300, or 1/1000. Dilutions of standard and plasma were in 0.1% BSA in TBST. After three washes, wells were incubated for 2 h with 50 μl of 1 μg/ml mouse anti‐human CRTAC1 monoclonal antibody (mAb), IgG_2b_, clone 755339 (catalog No. MAB52341) in TBST‐0.1% BSA. Wells were washed and incubated for 1 h with 50 μl of peroxidase‐conjugated rabbit anti‐mouse IgG (Jackson Immunoresearch) diluted 1/10,000 in TBST‐0.1% BSA. Wells were washed again and incubated with 50 μl KBL SureBlue TMB microwell peroxidase substrate (1‐component; SeraCare). The reaction was stopped with 50 μl KBL TMB stop solution (SeraCare). The absorbance of the colored product was measured at 450 nm, with wavelength correction at 620 nm, in a SpectraMax M5 plate reader (Molecular Devices). Each dilution of standard or unknown plasma was run in duplicate, the average of each duplicate unknown plasma was compared to the standard curve, and the values of dilutions of unknown plasma falling on the standard curve were averaged. An aliquot of pooled plasma collected from six healthy subjects was analyzed alongside the test samples to ensure the stability of the ELISA over time.

### 2.3 SDS‐PAGE and Western blot

Recombinant human CRTAC1 (2 μg in 8 μl phosphate‐buffered saline [PBS]) or human plasma (1 μl plus 7 μl PBS) was mixed with an equal volume of sample buffer (4% sodium dodecyl sulfate [SDS], 4 M urea, 5% glycerol, 62.5 mM Tris, pH 6.8 with bromophenol blue) and run under reducing conditions (10% β‐mercaptoethanol) on 4%–20% SDS‐polyacrylamide gel electrophoresis (PAGE) (Mini‐Protean TGX gels, Bio‐Rad). The gel was stained using Gelcode Blue (Thermo Fisher Scientific) or immunoblotted after transfer to polyvinylidene difluoride (PVDF) membranes using a Trans‐Blot Turbo mini PVDF Transfer Pack and Trans‐Blot Turbo Blotting System (Bio‐Rad). Membranes were blocked for 1 h at room temperature with 0.25% gelatin in TBST (TBST‐G). All the following steps were performed at room temperature. Membranes were incubated with primary anti‐CRTAC1 antibodies (polyclonal at 1 μg/ml or mAb at 2 μg/ml) in TBST‐G overnight, washed four times with TBST for 15 min and twice for 30 s, and incubated for 1 h with peroxidase‐conjugated anti‐sheep or anti‐mouse IgG (Jackson), 1/20,000, plus 2 μl Precision Protein StrepTactin‐HRP conjugate (BioRad) in TBST‐G. After washing, bands were detected with enhanced chemoluminescence (Clarity Western ECL substrate, BioRad). Specificity of the secondary antibodies was assessed by omitting the primary antibodies. Images were processed using the BioSpectrum 810 imaging system and VisionWorks LS software (UVP).

### 2.4 Curation of mass spectrometry data on plasma proteins in hospitalized COVID‐19 patients

For each of the 517 entries for proteins reported in our prior paper (Overmyer et al., 2021), mean log intensity‐based absolute quantification (IBAQ) score, log standard deviation (SD), and coefficient of variation (CV, log SD divided by log mean) for the 102 samples were calculated. Entries were queried in UniProt (https://www.uniprot.org/) according to accession number to confirm or assign UniProt entry names. In 15 instances we found two entries having the same entry name associated with different accession numbers. For each of the pairs, the one with the less well‐supported accession number had the smaller IBAQ score and higher CV and was discarded, leaving 502 unique entry names (Supplementary Spreadsheet 1). As indicated on the same spreadsheet, proteins were classified as cytoplasmic or being processed through ER and, if processed through ER, whether retained in ER or another cellular compartment, secreted into solution, deposited in extracellular matrix (ECM), or expressed on cell surfaces based on the descriptions in UniProt. Likely cells or tissues of origin were identified based on inspection of the top 10 mRNA‐expressing cells or tissues presented by GeneVisible (https://genevisible.com/) and single‐cell RNA data in the Human Protein Atlas (https://www.proteinatlas.org/). Acute phase proteins were labeled as positive or negative according to Table 1 of Gabay and Kushner (1999).

To learn which peptides of CRTAC1 were identified in patients' plasma, peptide identities were searched in data previously deposited (Overmyer et al., 2021) in MassIVE (https://doi.org/10.25345/C5F74G; accession number MSV000085703).

### 2.5 Interaction of CRTAC1 and CFP

A protein interaction ELISA was developed with the CRTAC1 reagents described above and recombinant human CFP lacking the signal peptide (residues 28‐469 with C‐terminal 10‐His tag, produced as a secreted protein by HEK293 cells, R&D catalog No. 8216‐PR‐050). Wells of 96‐well EIA/RIA flat bottom high‐protein binding capacity polystyrene microtiter plates (Corning) were coated overnight at 4°C with 50 μl of CFP, or BSA as control, in TBS. Subsequent steps were at room temperature. Solutions were decanted, wells were blocked for 1 h with 200 μl 1% BSA in TBST, and wells were washed three times with TBST. Wells were then incubated for 1 h with 50 μl CRTAC1 (or buffer as control) in TBST‐0.1% BSA. In some experiments, wells were instead incubated for 30 minutes with a mixture of CRTAC1 and CFP as a soluble competitor after the two proteins had been preincubated separately for 1 h. After three washes, wells were incubated for 2 h with 50 μl of 1 μg/ml of the mouse anti‐CRTAC1 mAb in TBST‐0.1% BSA. Wells were washed and incubated for 1 h with peroxidase‐conjugated anti‐mouse IgG 1/10,000 in TBST‐0.1% BSA. Wells were washed again and incubated with peroxidase substrate, the reaction was stopped, and the absorbance of the product was measured as in the ELISA above. Each condition was run in triplicate or duplicate.

### 2.6 Statistical analysis

Spearman rank test was used to analyze correlations with clinical data. To deal with heteroscedasticity in concentrations of plasma proteins, statistics were done using log CRTAC1 concentrations, and concentration data are plotted using log scales. Pearson test was used to analyze correlations of log 10‐transformed data. Analysis of variance (ANOVA) with Tukey's multiple comparisons posttest was used to compare log 10 data among three or more groups of subjects. Two‐tailed *t*‐test was used to compare log 10 data between two groups of subjects. Two‐tailed paired *t*‐test was used to compare log 10 data between two samples from the same subjects. A probability (*p*) ≤0.05 was considered significant. Analyses were done and graphs generated using Prism (GraphPad).

In addition, we evaluated the effects of patient age and various patient conditions using the following model:

$$y=\alpha +\mathrm{\beta x}+\sum_{i=1}^{7}\gamma_{i}\delta_{i}+\varepsilon$$

where *y =* log_10_(CRTAC1 concentration, nm), *α* is the expected value of *y* of a healthy control at age 0, *x* is the patient's age in years, *β* is the expected change in *y* for a 1‐year increase in age, *i* is one of the seven patient conditions, *γ*_*i*_ is the expected difference in the mean of *y* between condition *i* and the healthy control group, *δ*_*i*_ is an indicator variable, set to 1 if the patient has condition *i* and 0 otherwise, and *ε* is the error term. The seven conditions are “COPD,” “long COVID,” “long COVID + COPD,” “hospital, no COVID, no ICU,” “hospital, no COVID, ICU,” “hospital, COVID, no ICU,” and “hospital, COVID, ICU.” The analysis was performed in R (https://www.R‐project.org/). The emmeans (https://CRAN.R‐project.org/package=emmeans) library was used to assess statistical significance of selected contrasts. Our R markdown file and its HTML output are available on GitHub.

### 2.7 Study approval

Ethical approval was obtained from the Albany Medical College Committee on Research Involving Human Subjects (IRB# 5670‐20 and 4393‐20). Written informed consent was received prior to participation.

## 3 RESULTS

### 3.1 Characteristics and validation of an ELISA for CRTAC1 in human plasma

**FIGURE 1:**
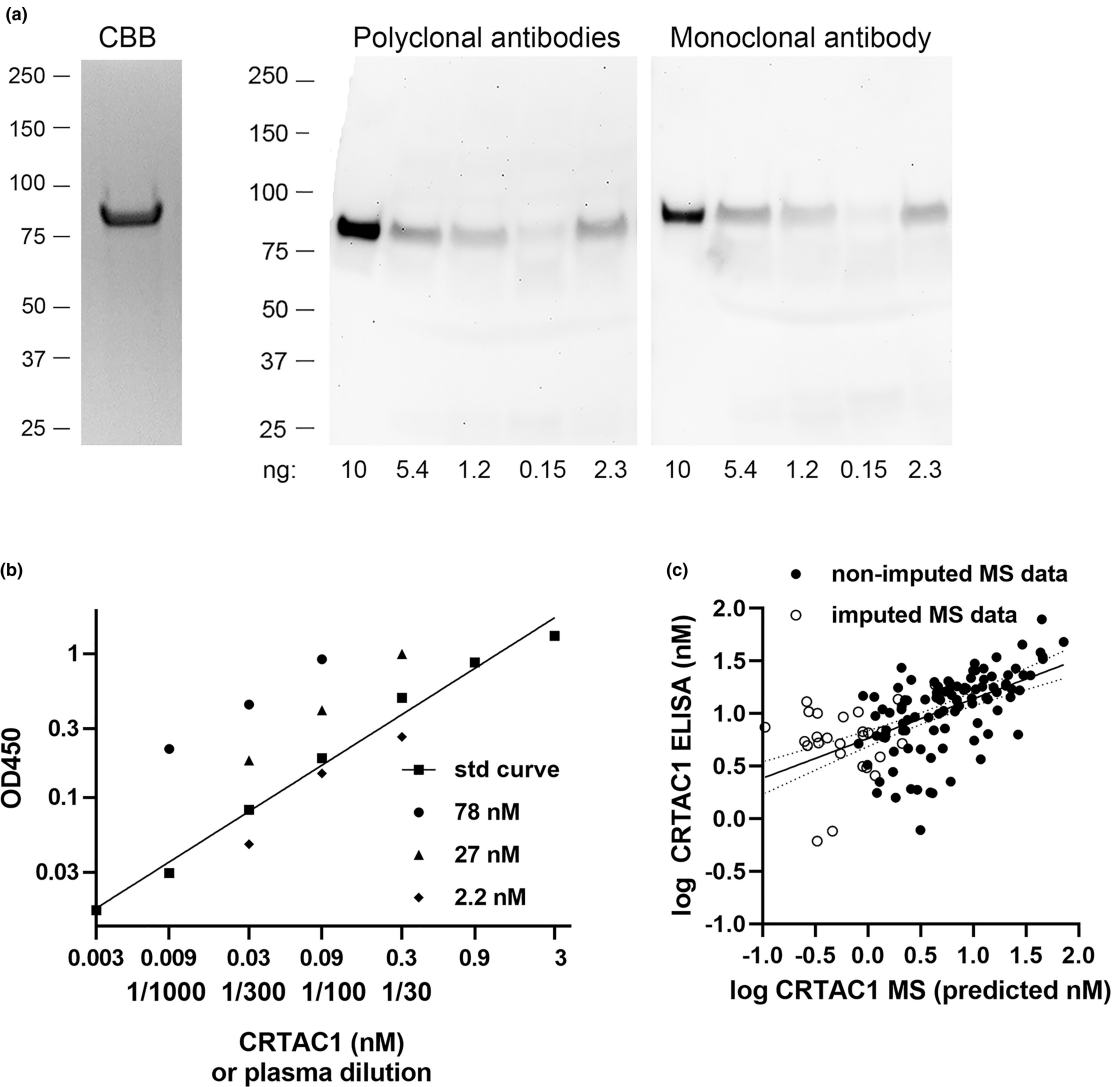
Western blot and development of ELISA for CRTAC1 and comparison of CRTAC1 concentrations in plasma as determined by ELISA and mass spectrometry. (a) Left: SDS‐PAGE Gelcode Blue staining of recombinant human CRTAC1 (2 μg/well) under reducing conditions; right: immunoblots under reducing conditions using sheep polyclonal antibodies or mouse mAb to recognize recombinant human CRTAC1 (10 ng) (lane 1) or CRTAC1 in four plasma samples (lanes 2–5) estimated to vary in amount of CRTAC1 by ELISA as indicated below the lane. Positions of molecular size markers (kDa) on the left. (b) Representative ELISA for human CRTAC1: optical density (OD) at 450 nm versus a standard (std) curve of recombinant human CRTAC1 in nM or dilutions of three plasma samples for which the indicated concentrations were determined. (c) CRTAC1 concentration (log nM) of plasma samples from hospitalized patients with or without COVID‐19 determined by ELISA versus concentration predicted by mass spectrometry (MS) compared to ALB as described in the text. Shown are the linear regression of the log_10_ values and 95% confidence intervals; closed circles, samples with non‐imputed MS data; open circles, samples with imputed MS data, *n* = 128 of which 27 had imputed MS data. Pearson correlation coefficient (*r*) for all samples = 0.58, probability (*p*) < 0.0001; for non‐imputed samples *r* = 0.58, *p* < 0.0001; and for imputed samples *r* = 0.16, *p* = 0.42.

Because close to a quarter of the estimates of plasma CRTAC1 concentration in our mass spectrometry‐based proteomic study of hospitalized patients were imputed (Overmyer et al., 2021), we developed a sensitive sandwich ELISA using sheep affinity‐purified polyclonal antibodies and mouse monoclonal antibody, respectively, to capture and detect human CRTAC1. The standard was commercial recombinant human CRTAC1 that ran as a single band with a nominal size of 87‐kDa on SDS‐PAGE under reducing conditions (Figure 1a, left) and was recognized in immunoblotting by both antibody populations (Figure 1a, right, lane 1). The antibodies recognized a band with a nominal size of 87‐kDa in plasma of COVID‐19 patients; the strength of this band matched the amount of CRTAC1 in the plasma as estimated by the ELISA (Figure 1a, right, lanes 2–5). No lower or higher apparent molecular mass bands were found, indicating that the predominant form of CRTAC1 in plasma is not modified by proteolysis or covalent crosslinking.

The standard curve for the double‐site ELISA was linear over a 1000‐fold range and allowed plasmas with low concentrations to be tested at 30‐fold and greater dilutions (Figure 1b). We carried out additional validation by comparing the nM concentrations determined by ELISA to those estimated from the IBAQ scores determined in our previous study (Overmyer et al., 2021). The IBAQ score is proportional to a protein's molar abundance (Cox et al., 2014). To estimate molar concentration from intensity‐based data, therefore, we multiplied the ratio of IBAQ scores of CRTAC1 and ALB (albumin) by the molar concentration of ALB, which was measured in the clinical laboratory close to the time of subject enrollment (Overmyer et al., 2021). Considering only the patients with non‐imputed IBAQ data, the methods correlate with *r* = 0.58 (*p* < 0.0001), whereas for only the patients with imputed IBAQ data, the methods do not correlate (*r* = 0.16, *p* = 0.42) (Figure 1c). The ELISA, therefore, extended the range over which concentrations can be quantified meaningfully from ~4 nM to below 0.6 nM and was capable of measuring molar concentrations of plasma CRTAC1 across the full range of values found in the patients.

### 3.2 Range of plasma CRTAC1 in normal individuals and patients with stable COPD

**FIGURE 2:**
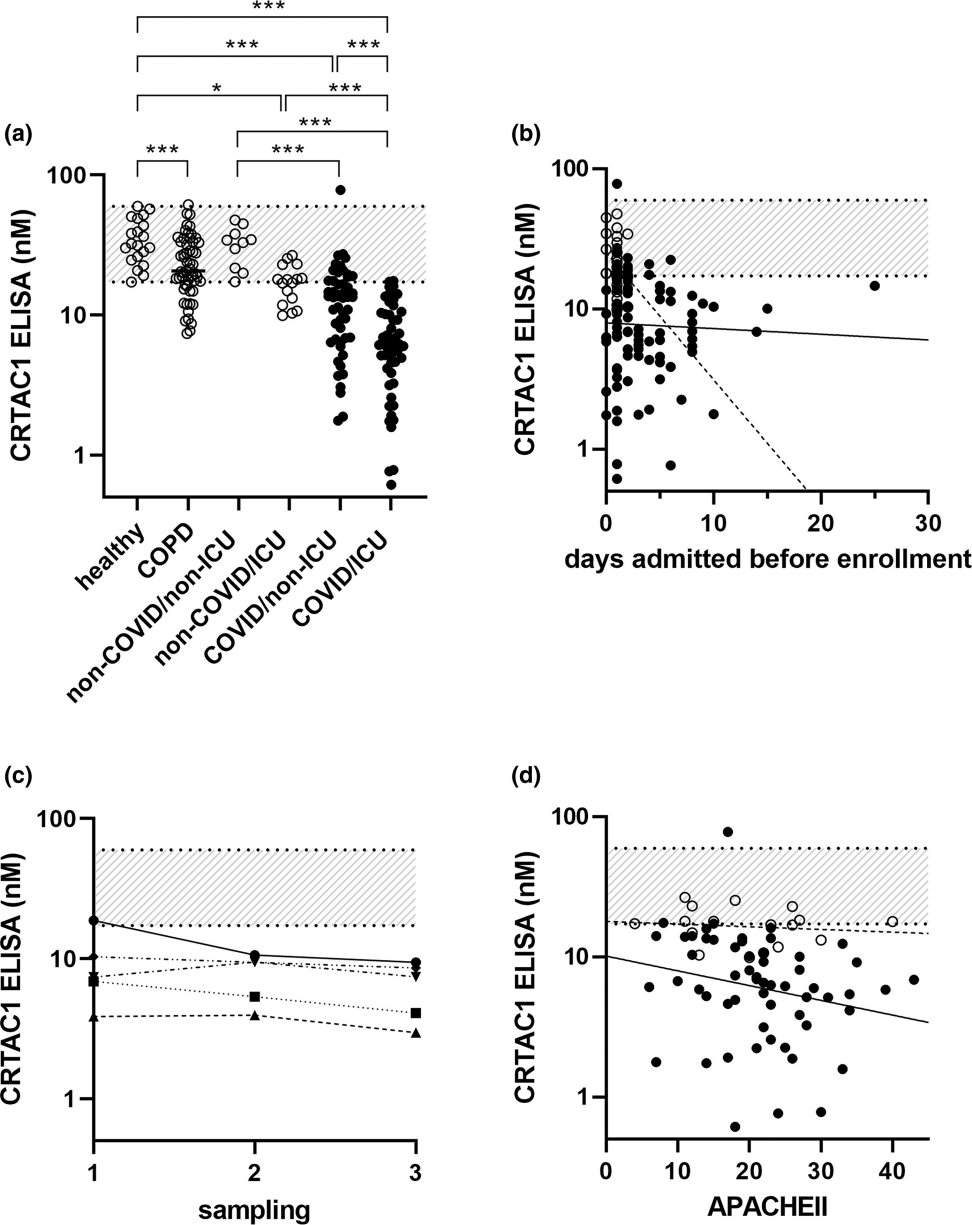
Plasma CRTAC1 concentrations determined by ELISA in different subject groups and relation to hospital day and clinical severity score. (a) The 20 healthy control subjects, 55 patients with COPD, and 128 hospitalized patients divided into groups without (*n* = 26, non‐COVID, open circles) or with (*n* = 102, COVID, closed circles) COVID‐19 and further divided into patients who were not (*n* = 10 + 51, respectively, non‐ICU) or were (*n* = 16 + 51, respectively, ICU) in the intensive care unit at time of enrollment. ***p ≤ 0.001, *p ≤ 0.02 (*t*‐test for pairwise comparison of COPD versus healthy, otherwise Tukey's multiple comparisons posttest). (b) CRTAC1 concentration versus day of hospitalization. The 128 patients divided into groups without (*n* = 26, open circles, dashed line) or with COVID‐19 (*n* = 102, closed circles, solid line). Spearman rank correlation coefficient (*r*_s_) for COVID‐19 = −0.10, *p* = 0.30; for non‐COVID‐19 *r*_s_ = −0.28, *p* = 0.16, for all *r*_s_ = −0.29, *p* = 0.0009. (c) CRTAC1 concentration in another set of hospitalized patients with COVID‐19 (*n* = 5) who were sampled more than once with 3‐day intervals. *D*: CRTAC1 concentration versus APACHE (acute physiological assessment and chronic health evaluation) II score. The 75 patients given an APACHEII score are divided into groups without (*n* = 17, open circles, dashed line) or with COVID‐19 (*n* = 58, closed circles, solid line). *r*_s_ for COVID‐19 = −0.33, *p* = 0.01; for non‐COVID‐19 *r*_s_ = −0.20, *p* = 0.44; for all *r*_s_ = −0.30, *p* = 0.009. Band, range of healthy subjects (17.2–59.7 nM).

The CRTAC1 concentration determined by ELISA in plasma from the 20 healthy subjects ranged from 17.2 to 59.7 nM with a mean of 35.5 nM and SD of 12.9 nM (Figure 2a). As a comparison group at increased risk for severe COVID‐19, we analyzed CRTAC1 in samples obtained from 55 older patients with stable COPD who had no history of COVID‐19. Concentrations ranged from 7.4 to 61.3 nM with a mean of 24.6 nM and SD of 12.3 nM (Figure 2a). Values in COPD patients overlapped but differed significantly from those of healthy individuals (*p* = 0.001).

### 3.3 CRTAC1 concentration in non‐COVID‐19 and COVID‐19 patients receiving hospital care for respiratory distress

ELISAs were done on plasmas from the previously studied (Overmyer et al., 2021) 128 adult patients admitted for moderate to severe respiratory issues requiring supplemental oxygen who tested positive (*n* = 102) or negative (*n* = 26) for SARS‐CoV2. Of patients without COVID‐19, 0% of those not admitted to the ICU and 38% of those admitted to the ICU had levels <17 nM. In contrast, of patients with COVID‐19, 69% of those not admitted to the ICU and 96% of those admitted to the ICU had levels <17 nM (Figure 2a). The concentrations in the COVID‐19 patients in the ICU were significantly lower than those of the other three groups (Figure 2a). Three values were <1 nM, that is, <3% of the normal mean or <6% the lower limit of normal, the lowest being 0.6 nM or 2% of the normal mean. Values in COVID‐19 patients not in the ICU were similar to patients without COVID‐19 who were in the ICU. Comparing patients not in the ICU, those with COVID‐19 had significantly lower levels than those without COVID‐19. There was no effect of age in the comparisons among the four groups of hospitalized patients (Supplementary information on Github).

### 3.4 Changes during hospitalization

Most of the patients were enrolled in the study during the first 2 days of hospitalization, but some were enrolled later. CRTAC1 concentrations were low in the samples obtained in patients enrolled early or later with no indication of an upward trend (Figure 2b). To test for changes in CRTAC1 concentration during hospitalization, five patients with COVID‐19 who were not among the 102 hospitalized COVID‐19 patients described above were studied at 3‐day intervals; all had below normal concentrations that varied little (Figure 2c). These results indicate that low CRTAC1 concentration is a stable finding during prolonged hospitalizations for COVID‐19.

### 3.5 Correlations between CRTAC1 and clinical severity scores and laboratory biomarkers in hospitalized COVID‐19 patients

Plasma CRTAC1 concentration correlated inversely with acute physiological assessment and chronic health evaluation (APACHE) II, sequential organ failure assessment (SOFA), simplified acute physiology score (SAPS) II, and World Health Organization (WHO) scores of disease or COVID‐19 severity 19 (Izcovich et al., 2020; Wilfong et al., 2021) and directly with number of ventilation‐free or hospital‐free days (HFD‐45) through Day 45 after entry, that is, with speed of recovery and discharge from the hospital (Table 2 and Figure 2d). CRTAC1 was significantly lower in patients who were on mechanical ventilation at the time of entry in the study (median 5.8 nM, range 0.6 to 17 nM, *n* = 43) than in those who were not (median 13 nM, range 1.8 to 78 nM, *n* = 59) (*p* < 0.0001). All COVID‐19 patients with HFD‐45 = 0 had CRTAC1 < 17 nM, that is, below or at the bottom of the range of healthy subjects. However, two of the three patients with the lowest concentrations survived, suggesting that there is no threshold below which CRTAC1 concentration portends inability to recover.

**TABLE 2:** Correlations between plasma CRTAC1 concentration determined by ELISA and various variables in the hospitalized patients with COVID‐19.

Variable	*r*_s_	*p*	FDR	FWER	*n*
Age	0.07	0.51	0.51	1	102
Severity indices					
Charlson comorbidity index	0.08	0.43	0.45	1	102
APACHEII	−0.33	0.01	0.019	0.23	58
SOFA	−0.30	0.02	0.031	0.46	57
SAPSII	−0.35	0.007	0.015	0.16	57
Vent‐free days	0.37	0.0001	0.001	0.0023	102
HFD‐45	0.35	0.0004	0.002	0.0092	102
WHO	−0.32	0.001	0.003	0.023	102
Biomarkers					
Ferritin	−0.21	0.04	0.058	0.92	96
CRP	−0.30	0.003	0.008	0.069	94
D‐dimer	−0.32	0.002	0.006	0.046	87
Procalcitonin	−0.36	0.0006	0.003	0.014	89
Lactate	−0.11	0.40	0.44	1	65
Fibrinogen	−0.18	0.10	0.12	1	81
Albumin	0.34	0.0004	0.002	0.0092	102
Hemogram					
WBC	−0.23	0.02	0.031	0.46	102
Hemoglobin	0.24	0.02	0.031	0.46	102
Mean corpuscular volume	0.19	0.06	0.081	1	102
Platelets	−0.13	0.20	0.23	1	102
Neutrophils	−0.33	0.0008	0.003	0.019	102
Lymphocytes	0.28	0.004	0.009	0.092	102
Monocytes	0.38	0.0001	0.001	0.0023	102
Eosinophils	0.18	0.07	0.089	1	102

Abbreviations: APACHE, acute physiological assessment and chronic health evaluation; CRP, C‐reactive protein; FDR, Benjamini and Hochberg false discovery rate (Benjamini & Hochberg, 1995); FWER, Bonferroni family‐wise error rate (Bland & Altman, 1995); HFD‐45, hospital‐free days at Day 45; *n*, number of patients; *p*, probability; *r*_s_, Spearman rank correlation coefficient; SAPS, simplified acute physiology score; SOFA, sequential organ failure assessment; vent, ventilator; WBC, white blood cells; WHO, World Health Organization.

CRTAC1 correlated directly with the clinical laboratory measurements of ALB, a negative acute phase protein; and inversely with measurements of CRP (C‐reactive protein), the paradigm positive acute phase protein; fibrin D‐dimer; and procalcitonin (Table 2). CRTAC1 correlated directly with hemoglobin and lymphocyte and monocyte counts and inversely with total white blood cell and neutrophil counts (Table 2).

### 3.6 CRTAC1 concentration after recovery from COVID‐19

**FIGURE 3:**
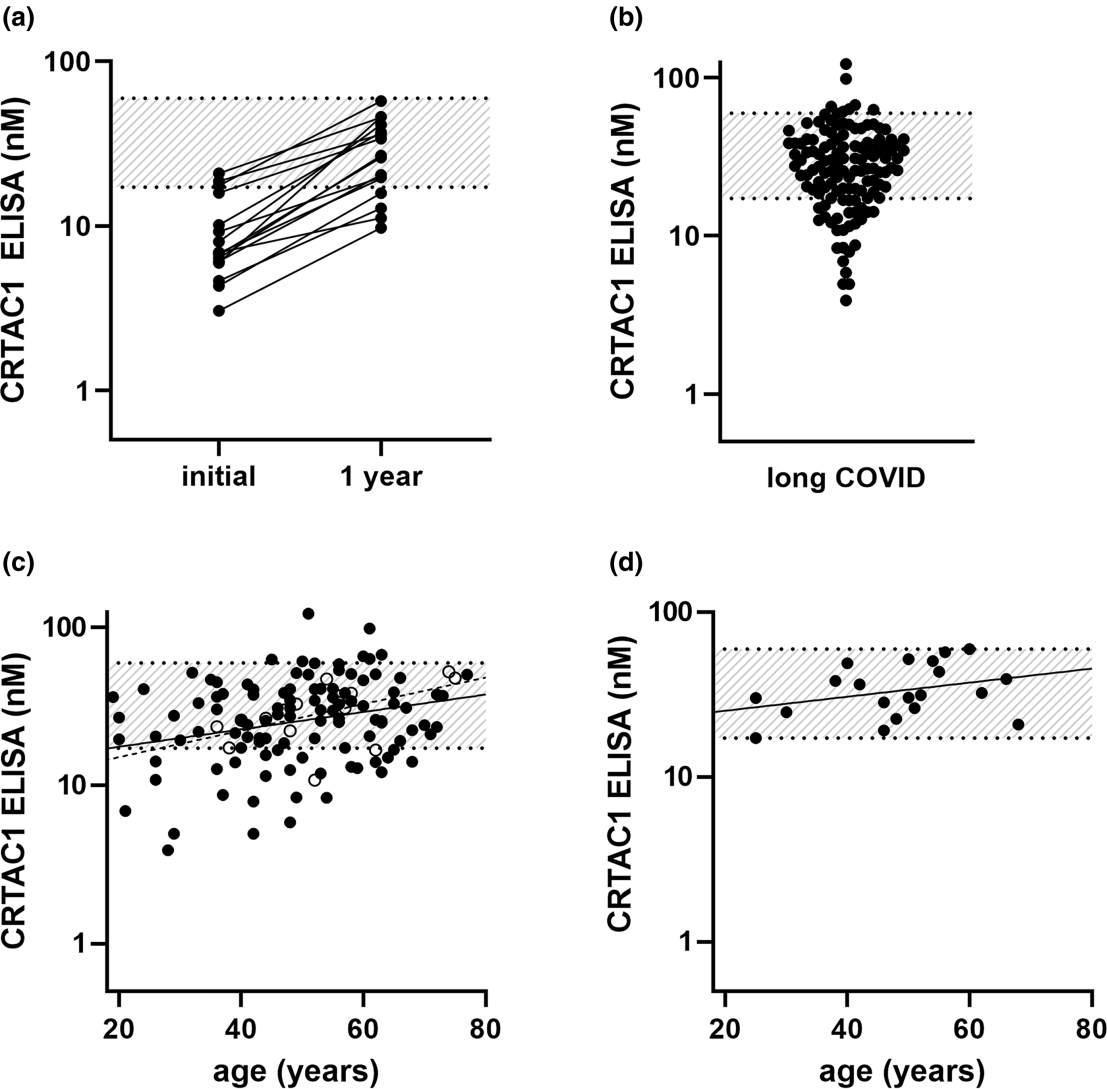
Plasma CRTAC1 concentrations determined by ELISA in patients after COVID‐19 treated in or outside the hospital. (a) A subset (*n* = 16) of the 102 patients hospitalized with COVID‐19 were sampled 1 year after hospitalization, *p* < 0.0001 for a year later (1 year) versus the time of hospitalization (initial) (paired t test of log_10_ data). (b) Patients with long COVID (*n* = 127); *p* = 0.02 for long COVID versus healthy and *p* = 0.008 for long COVID versus COPD (*t*‐test). (c) Plot of plasma CRTAC1 versus age in long COVID patients without (*n* = 111, closed circles, solid line) and with (*n* = 16, open circles, dashed line) COPD, with linear regressions. (d) Plot of plasma CRTAC1 versus age in healthy controls (*n* = 20) with linear regression. Band, range of healthy subjects (17.2–59.7 nM).

Previously, it was reported that plasma CRTAC1 fell from pre‐illness levels in five critically ill COVID‐19 patients as estimated by tandem mass tagging (Byeon et al., 2022). To learn if the reverse is true and CRTAC1 concentration recovers after severe COVID‐19, we consented 16 patients from our cohort of 102 (Overmyer et al., 2021) to be studied a year after hospitalization. CRTAC1 increased in all (Figure 3a). However, four (25%) had CRTAC1 concentrations that remained below normal.

The four low values prompted us to study 127 patients who suffered from long COVID after COVID‐19 that did not require hospitalization. For the group as a whole, mean concentration was 31 nM with SD of 18 nM (Figure 3b; Supplementary information deposited on Github). Although the mean of the long COVID group was only marginally different from the healthy controls, the distribution was, on the log_10_ scale, much broader, negatively skewed, and deviating significantly from normality according to the Shapiro–Wilk test. Values tailed to as low as 4 nM (Figure 3b), not as low as the lowest values in patients hospitalized with respiratory distress and COVID‐19 but lower than the lowest values in patients who were hospitalized with respiratory distress not due to COVID‐19 or patients with stable COPD (Figure 2a). Values <17 nM were found in 24 patients (19%) (Figure 3b). Compared to healthy controls, 11 patients (9%) were outliers with Z scores <−3, whereas zero such patients would be expected if the two distributions were identical. Thus, there was a subset of long COVID patients with CRTAC1 concentrations below the range of healthy subjects.

Analysis of the 102 long COVID patients for whom clinical data were available revealed no significant correlations between CRTAC1 concentrations and forced ventilatory capacity, forced expiratory volume‐1 s, body mass index, or domains or composite score of Short Form 36 Health Survey (SF‐36), and no significant differences in sex or presence/absence of diabetes between the patients with CRTAC1 < 17 nM and patients with concentrations >17 nM (Supplementary spreadsheet 2 and matrix plot). A direct correlation with age (Spearman rank correlation coefficient [*r*_s_] = 0.24, *p* = 0.006; Pearson correlation coefficient [*r*] for log CRTAC1 = 0.28, *p* = 0.001) was found for patients with long COVID (Figure 3c). A similar direct correlation with age was found in 37,278 Icelandic individuals for CRTAC1 measured by the dimensionless slow off‐rate modified aptamer (SOMAscan) assay (Styrkarsdottir et al., 2021), and a trend to a correlation with age was found for our limited number of healthy subjects (*r*_s_ = 0.37, *p* = 0.11; *r* = 0.32, *p* = 0.16) (Figure 3d). In spite of these correlations and the statistical significance of the age coefficient in the model described in the Methods, the age effect on CRTAC1 concentration was relatively small and did not account for the presence of long COVID patients with abnormally low values.

Long COVID patients who had COPD comorbidity had CRTAC1 values that were similar to the long COVID group as a whole (Figure 3c; Supplementary information Github info). Furthermore, 10 of the 11 patients with CRTAC1 z scores <−3 did not have COPD. Thus, COPD comorbidity also did not explain the existence of the long COVID outliers with CRTAC1 levels below normal.

### 3.7 Possible role of CFP in depressing concentration of plasma CRTAC1 in severe COVID‐19

**FIGURE 4:**
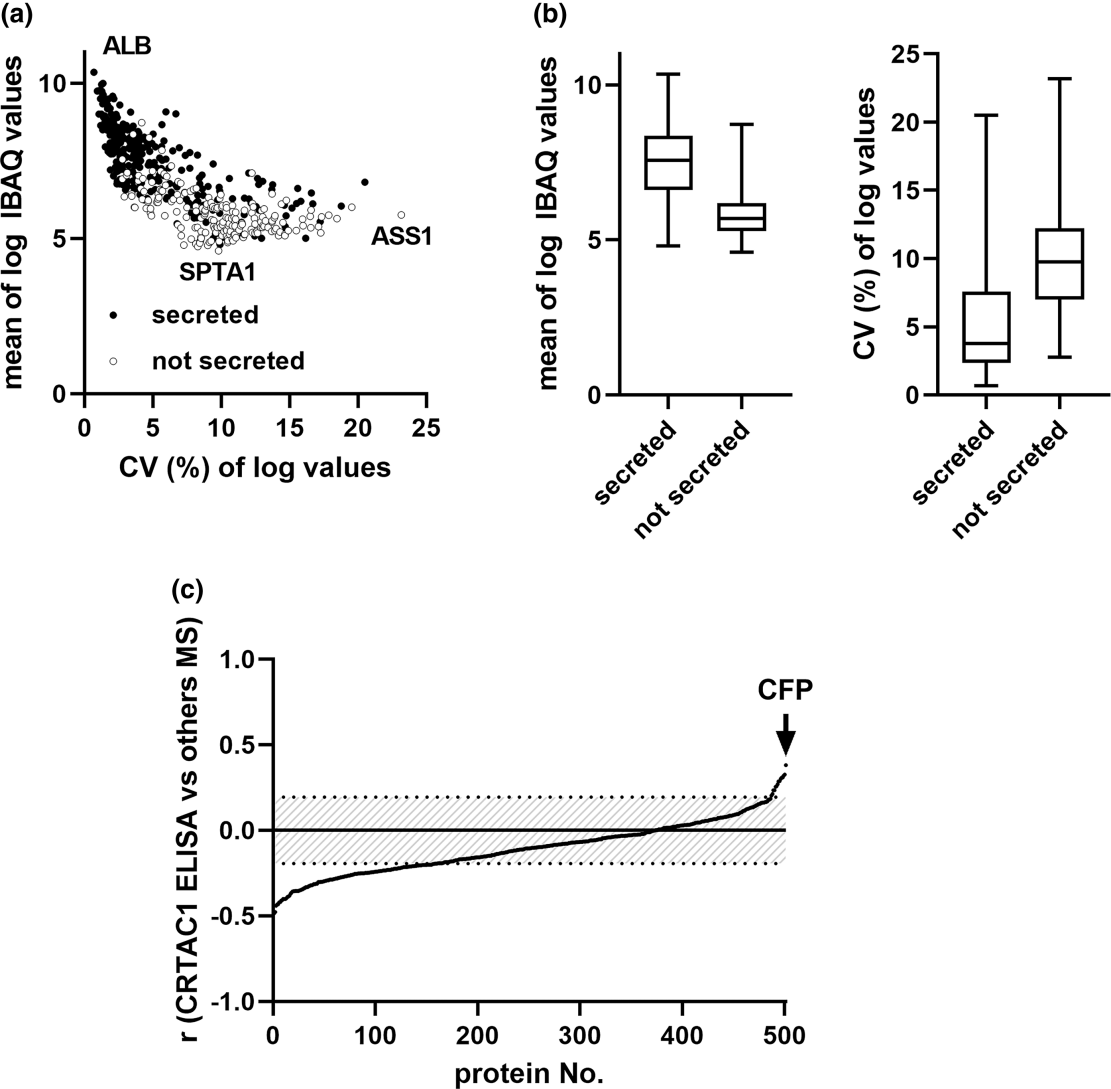
IBAQ scores and CVs of plasma proteins in hospitalized COVID‐19 patients. (a) Scatter plot of means of log_10_ IBAQ value versus coefficients of variation (CVs) calculated based on log_10_ IBAQ scores of the 501 proteins. Closed circles, secreted proteins; open circles, non‐secreted proteins. The proteins with the highest and lowest IBAQ scores (ALB and SPTA1, respectively) and one outlier (ASS1) are indicated. (b) Box plots (boxes representing medians and quartiles, whiskers representing minimum and maximum) of means of log_10_ IBAQ scores (left) and CVs (right) of secreted and not secreted proteins, *p* < 0.0001 for each comparison. (c) Pearson correlation coefficient (*r*) between log_10_ CRTAC1 determined by ELISA and log_10_ IBAQ value by mass spectrometry (MS) of the 501 plasma proteins. *p* for *r* outside the band <0.05.

We compared the concentrations of CRTAC1 as determined by ELISA and other circulating proteins as determined by IBAQ scores with the goal of finding commonalities that might explain depressed plasma CRTAC1 in severe COVID‐19. We classified the circulating proteins regarding likely route of release into plasma, likely cellular source, and whether the protein is a recognized positive or negative acute phase protein (Supplementary Spreadsheet 1). Of the 501 other proteins, 304 (61%) are annotated as being secreted through the ER into plasma. Of the remaining 197, 154 are annotated as cytoplasmic or resident ER proteins, 33 as cell surface proteins, and 9 as ECM proteins. We captured patient‐to‐patient variation in levels of a single protein in the COVID‐19 patients as log_10_‐based coefficients of variation (CVs), that is, per cent of standard deviation of the log_10_ IBAQ scores divided by mean log_10_ IBAQ value (Supplementary Spreadsheet 1). Log_10_ IBAQ scores ranged from 10.4 for ALB to 4.6 for SPTA1, and log_10_ CVs ranged from 0.7% for ALB to 23.2% for ASS1 (Figure 4a). CVs correlated with log_10_ IBAQ scores (*r*_s_ = −0.82, *r* = −0.76, *p* < 0.0001). Secreted proteins as a group were significantly more abundant and less variable than proteins released into plasma by other routes (Figure 4b). Similar abundance distributions of secreted proteins and non‐secreted proteins were reported for normal human plasma by Geyer et al. (2016).

Of 158 proteins with significant (*p* < 0.05) inverse correlations with CRTAC1 (Figure 4c), 59%, including the 13 with the highest correlations, are not secreted classically through ER as soluble proteins and would require cell breakage or perturbation. Nearly all non‐classically secreted proteins (93 of 94) have log_10_ CVs >3.8. Of the inversely correlated proteins secreted through ER as soluble proteins, 50% (32/54) have log_10_ CVs <3.9. Inversely correlated classically secreted proteins include eight positive acute phase proteins (SERPINA3, SERPINA1, ITIH4, SAA2, SAA1, CP, ORM1, CD163); nine complement components (CPN1, C9, SERPING1, C6, CFHR5, CPN2, CFB, FCN2, C5); four coagulation proteins (F5, SERPINF2, F9, F11); and 21 immunoglobulin segments, including IGHV4‐34 and IGHV1‐69 that are found in autoantibodies and increased in children with multisystems inflammatory syndrome associated with COVID‐19 (Porritt et al., 2021; Supplementary Spreadsheet 1). The mix of proteins with significant inverse correlations indicate that decreases in CRTAC1 are linked to processes that result in greater cell disruption, acute phase protein response, upregulation of proteins of the complement and coagulations systems, and virally induced production of immunoglobulins.

Only 15 proteins had significant direct correlations with CRTAC1 (Figure 4c). Of the 15 proteins that correlated directly, 13 are secreted through ER like CRTAC1—ALB, CFP, PGLYRP, ECM1, PI16, GSN, CLEC1B, HGFAC, APOD, TTR, GPLD1, F13B, and AHSG (GSN exists in cytoplasmic and secreted forms due to alternative splicing; we previously demonstrated that only the secreted form is decreased in plasma in severe COVID‐19 (Overmyer et al., 2021)). Cells would need to break or be perturbed for the remaining proteins with a significant direct correlation—AIFM1, a mitochondrial protein, and CHL1, a neural cell surface protein—to enter the circulation. The log CVs of all except AIFM1, CHL1, and PI16 are much smaller than CRTAC1's 10.6% (Supplementary Spreadsheet 1). ALB and TTR are recognized acute phase proteins that decrease during inflammation due to decreased hepatocyte synthesis (Gabay & Kushner, 1999). Of the other directly correlating secreted proteins, eight are products of liver, CFP is a product of monocyte/macrophages (Boussier et al., 2022), and five are products of many cell types (Supplementary Spreadsheet 1).

Decreased plasma CRTAC1 could result from decreased production as occurs with ALB and TTR, increased turnover as occurs during severe infection with GSN serving as a scavenger for filamentous actin being released from cells (Piktel et al., 2018), or a combination of mechanisms. Analysis of cultured T2AE cells (Ballard et al., 2010) and single‐cell transcriptomic and proteomic data from pulmonary fibrosis patients (Mayr et al., 2021) and recent human expression atlases (Tabula Sapiens Consortium et al., 2022; Travaglini et al., 2020) indicate that T2AE cells are a major source of CRTAC1. Loss or de‐differentiation of T2AE cells is hypothesized to explain the decrease in plasma and bronchoalveolar lavage fluid CRTAC1 in pulmonary fibrosis (Mayr et al., 2021). However, for changes in T2AE cells to manifest as a many‐fold reduction in plasma CRTAC1 in severe COVID‐19, T2AE cells would need to be overwhelmingly the major source of CRTAC1 found in the circulating pool. Joint tissues contribute significantly to plasma CRTAC1 concentration as demonstrated by population‐wide proteomic studies that associate higher plasma CRTAC1 concentrations with severity and progression of osteoarthritis (Styrkarsdottir et al., 2021, 2023; Szilagyi et al., 2023). Indeed, in a small intensity‐based mass spectrometric study, a 5.5‐fold increase in CRTAC1 was found in osteoarthritis patients versus controls (Tardif et al., 2022). In addition, CRTAC1 production by T2AE cells would need to be lost almost completely to explain the extremely low CRTAC1 levels. Fewer T2AE cells were found in fatal COVID‐19 cases, even at early stages before typical patterns of acute lung injury were apparent histologically (Chait et al., 2022; Delorey et al., 2021); and single‐cell sequencing of lungs from patients with fatal COVID‐19 revealed major remodeling in the lung epithelial compartment and failed tissue regeneration dominated by defective T2AE cell differentiation (Delorey et al., 2021; Melms et al., 2021). However, SARS‐CoV2‐infected stem cell‐derived T2AE cells had increased rather than decreased CRTAC1 message compared to uninfected controls (Huang et al., 2020), and RNA‐Seq data in reference (Delorey et al., 2021) analyzed by the pseudobulk method revealed only a 1.3‐fold decrease in CRTAC1 mRNA in T2AE cells in lungs of deceased COVID‐19 patients compared to controls dying with healthy lungs (Supplementary spreadsheet 3). Accordingly, it is difficult to explain the profound decrease in concentration of circulating CRTAC1 based solely on problems with CRTAC1 production by T2AE cells.

**FIGURE 5:**
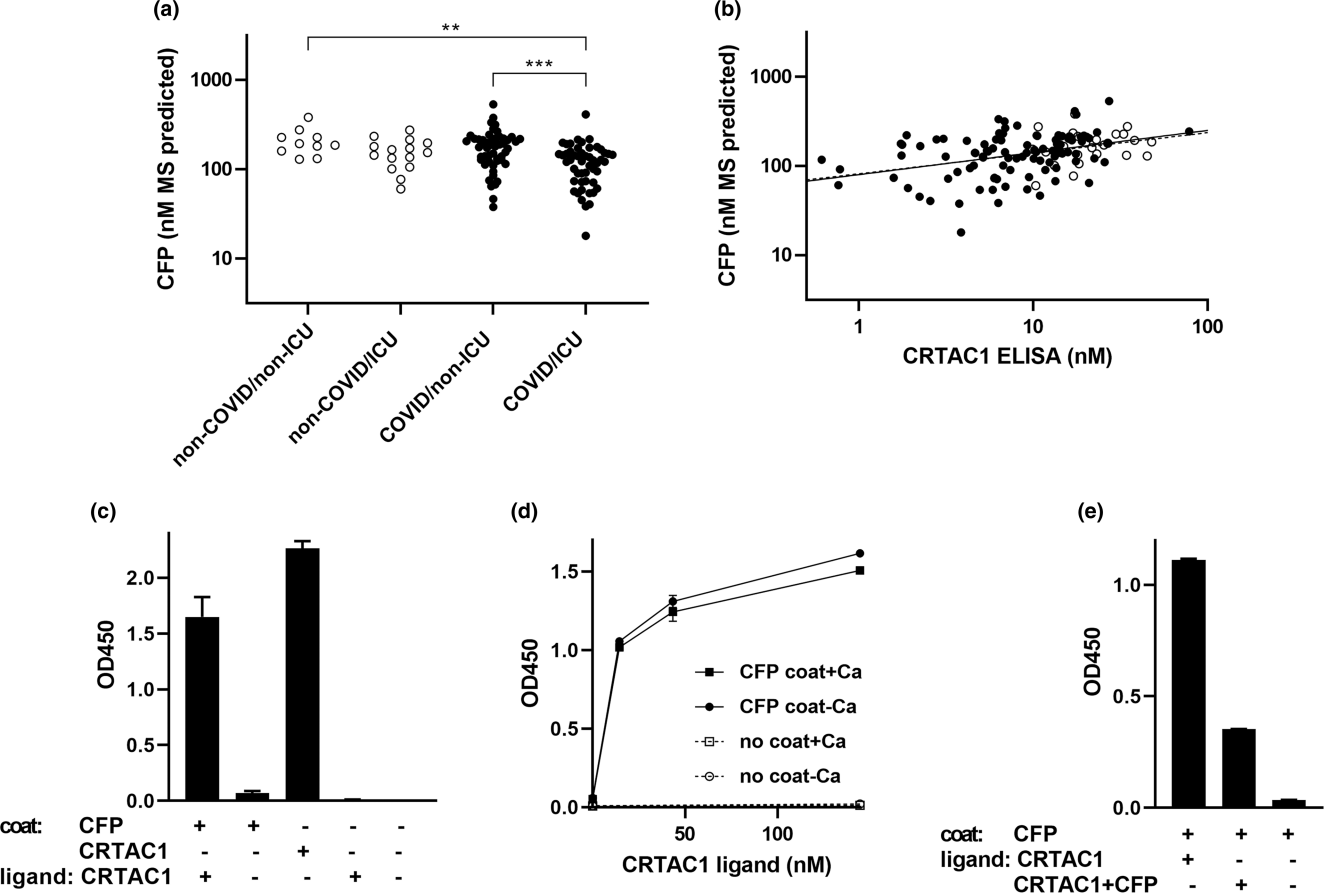
Interaction of CRTAC1 and CFP. (a) CFP concentrations predicted by ratio of IBAQ values with ALB in the hospitalized patients divided into groups without or with COVID‐19 and further divided into patients who were not or were in the intensive care unit at time of enrollment, as in Figure 2a. For comparisons of groups: ***p ≤ 0.001, **p ≤ 0.005 (Tukey's multiple comparisons posttest). (b) Scatter plot of nM concentrations of CFP predicted by ratio of IBAQ values with ALB and CRTAC1 determined by ELISA. Pearson correlation coefficient (*r*) for COVID‐19 (closed circles, solid line) = 0.37, *p* = 0.0001; for non‐COVID‐19 (open circles, dashed line) *r* = 0.26, *p* = 0.22. (c) Binding of 140 nM CRTAC1 (10 μg/mL) to immobilized CFP (coated at 200 nM [10 μg/mL]) as detected with anti‐CRTAC1. (d) Binding as detected with anti‐CRTAC1 of increasing concentrations of CRTAC1 in the absence or presence of 1 mM CaCl_2_ to immobilized CFP coated at 200 nM in TBS. (e) Inhibition by preincubation with 200 nM soluble CFP of binding of 43 nM CRTAC1 (3 μg/mL) to CFP coated at 60 nM (3 μg/mL); bound CRTAC1 detected with anti‐CRTAC1. Mean and standard error of the mean (SEM) of triplicate (c and e) or duplicate (d) wells.

Based on our finding that the highest direct correlation of CRTAC1 concentration is with IBAQ values for CFP (Figure 4c) and the identification by Huttlin et al. of a CRTAC1‐CFP interaction in a global screen of protein–protein interactions (Huttlin et al., 2021), we evaluated the hypothesis that plasma CRTAC1 is consumed along with plasma CFP during the intense activation of the alternate complement pathway that accompanies severe COVID‐19 ((Boussier et al., 2022; Siggins et al., 2023) and references therein). Others have used ELISAs to demonstrate that activation of the alternative complement pathway and decreased levels of CFP correlate with severity and mortality of COVID‐19 (Boussier et al., 2022; Siggins et al., 2023). In addition, deceased CFP was found to be accompanied by increased CFP mRNA expression in peripheral blood (Boussier et al., 2022), suggesting increased turnover of CFP that exceeds increased synthesis. To be able to compare molar concentrations of CFP and CRTAC1, we multiplied the ratio of IBAQ values of CFP and ALB by the independently determined molar concentration of ALB. The estimated concentrations of CFP in the range of 20‐ to 300‐nM range in Figure 5a,b are similar to the concentration ranges of CFP estimated by ELISA in published studies of COVID‐19 (Boussier et al., 2022; Siggins et al., 2023). CFP concentrations in the four hospitalized groups (Figure 5a) were higher and more clustered than the concentrations for CRTAC1 (Figure 2a) but had the same trend. CFP concentrations in patients in the ICU with COVID‐19 were significantly lower than patients not in the ICU. The direct correlation between the two concentrations was significant for COVID‐19 patients (*r* = 0.37, *p* = 0.0001) and trended toward significance in the smaller number of non‐COVID‐19 patients (*r* = 0.26, *p* = 0.22) (Figure 5b).

In the global screen revealing an interaction between CFP and CRTAC1, the two proteins were in 293 cell lysates, and affinity‐tagged CRTAC1 served as a bait (Huttlin et al., 2021). Prior to secretion, CFP and CRTAC1 are processed in the endoplasmic reticulum and Golgi to undergo disulfide bonding and C‐linked mannosylation, O‐linked fucosyl glucosylation, and N‐linked glycosylation in the case of CFP and disulfide bonding and O‐linked glycosylation in the case of CRTAC1 (Pedersen et al., 2023; Steck et al., 2007). To exclude the possibility that the interaction detected in cell lysate (Huttlin et al., 2021) is an artifact of incomplete protein processing, we set up an interaction ELISA to test interactions between recombinant CRTAC1 and CFP purified after secretion from mammalian cells with the required processing machinery. Soluble CRTAC1, 140 nM, bound to surfaces that had been coated with 200 nM CFP but not to control ALB‐blocked surfaces (Figure 5c). Specific binding of CRTAC1 at a concentration of 14 nM was found to surfaces coated with CFP at a concentration as low as 20 nM (not shown). CRTAC1 bound from solution to substrate‐coated CFP in both the absence and presence of Ca^2+^ (Figure 5d). Binding of 43 nM soluble CRTAC1 to CFP that had been immobilized at a concentration of 60 nM was inhibited by preincubation with 200 nM soluble CFP (Figure 5e). These results demonstrate an interaction between CRTAC1 and CFP at the nanomolar concentrations present in plasma and support the hypothesis that plasma CRTAC1 is consumed in severe COVID‐19 due to its interaction with CFP deposited to drive the alternate complement pathway.

## 4 DISCUSSION

Large‐scale multi‐omic studies such as we carried out on patients with severe COVID‐19 (Overmyer et al., 2021) have been indispensable in defining the broad molecular‐level reorganization that characterizes the host COVID‐19 viral response. Such studies also uncover changes worthy of validation by other methods and further explication. Such is the case for CRTAC1, which fell from an unknown normal concentration to a level that in many patients was undetectable by mass spectrometry (Overmyer et al., 2021). Here we report using a sensitive sandwich ELISA to determine plasma CRTAC1 concentration in our original cohort of patients hospitalized for respiratory distress during the first weeks of the COVID‐19 pandemic, healthy controls, COPD, and patients recovering from COVID‐19. We found that patients receiving treatment for COVID‐19 in an ICU almost uniformly had concentrations below the range found in healthy individuals, falling to as low as 2% of the normal mean. CRTAC1 concentration correlated with metrics of COVID‐19 severity and concentrations of many other plasma proteins, most notably CFP important for alternate complement pathway activation. One year after hospitalization, CRTAC1 increased uniformly although it did not normalize in all patients. A subset of long COVID patients also had below normal plasma CRTAC1. The results suggest that CRTAC1 levels do not return to normal in some patients recovering from both non‐severe and severe COVID‐19. In this discussion, we relate what is known about CRTAC1 in other contexts to the changes found in COVID‐19 and present speculative models to explain the changes.

CRTAC1 was identified in a subtractive hybridization screen for mRNAs present in chondrocytes and not in osteoblasts or mesenchymal stem cells (Steck et al., 2001). Subsequent studies demonstrated nonneural and neural proteoforms of CRTAC1 arising from differential splicing with expression of the nonneural proteoform in lung as well as cartilage (Steck et al., 2007). Nonneural human CRTAC1 is synthesized as a 661‐residue protein comprising a 27‐residue N‐terminal signal peptide, 372‐residue stretch in which four FG‐GAP motifs are embedded, 70‐residue UnbV‐ASPIC domain (UnbV refers to proteins in *Rhodopirellula baltica* and other bacteria, and ASPIC is an alternate name for CRTAC1 (Anjos et al., 2017; Redruello et al., 2010)), 47‐residue calcium‐binding EGF‐like domain, and low complexity 56‐residue C‐terminal segment that is subject to O‐glycosylation ((Steck et al., 2007), https://www.uniprot.org). The various motifs and domains are predicted to form a well‐structured globule to which an unstructured C‐terminal segment is appended (https://alphafold.ebi.ac.uk/entry/Q9NQ79).

The most functional information is known about the neural proteoform, which has a C‐terminal segment that ends in a presumptive transmembrane sequence. The neural proteoform was identified independently as lateral olfactory tract usher substance (LOTUS) in a fluorochrome‐assisted light‐inactivation screen for molecules that promote axonal growth cone extension in the olfactory tract (Sato et al., 2011). A knockout of LOTUS/CRTAC1 results in mice that have de‐fasciculation of the lateral olfactory tract and altered synaptic density in the hippocampus and memory formation (Nishida et al., 2021; Sato et al., 2011). Neural CRTAC1 binds to and antagonizes reticulon‐4 receptor (RTN4R), a leucine‐rich repeat protein with a lipid membrane anchor (Kurihara et al., 2014). RTN4R inhibits axon growth and regeneration in concert with RTN4 and other agonists. An engineered version of neural CRTAC1 lacking the C‐terminal transmembrane segment promotes growth cone extension but via a mechanism that blocks interaction of RTN4R with NGFR, a receptor for several neural growth factors (Kawakami et al., 2018). Nonneural circulating CRTAC1 presumably has the same activity as truncated neural CRTAC1 but to connect low CRTAC1 levels to CNS symptoms in COVID‐19 such as anosmia, it would need to be shown that the nonneural form crosses the blood–brain barrier and contributes functionally to the pool of CRTAC1 regulating neuronal connections. None of the proteins in interacting networks described for neural CRTAC1 contains the thrombospondin type 1 domains that comprise the bulk of CFP (https://www.uniprot.org/), suggesting that interactions of CRTAC1 with CFP and neural partners may utilize different interaction sites on CRTAC1.

We can only speculate about mechanisms that cause low circulating CRTAC1 in acute severe COVID‐19 and failure of CRTAC1 to return to normal in some patients after resolution of acute COVID‐19. CRTAC1 likely enters alveolar capillaries upon secretion from the basilar surface of T2AE cells and diffusion across alveolar basal lamina. Circulating CRTAC1 also originates from cells, most notably chondrocytes, outside of the lung. In severe COVID‐19, the contribution of T2AE cells to circulating CRTAC1 likely is impacted by loss of the cells per se (Chait et al., 2022; Delorey et al., 2021) and remodeling of the lung epithelial compartment dominated by de‐differentiation of T2AE cells with partial loss of CRTAC1 expression (Delorey et al., 2021; Melms et al., 2021). Proteomic analysis of plasma and BAL CRTAC1 of patients with idiopathic pulmonary fibrosis identified CRTAC1 as one of a small set of circulating proteins that is enriched in BAL when compared to plasma (Mayr et al., 2021). The enrichment suggests that basilar secretion from T2AE cells to capillary is impaired when T2AE cells undergo de‐differentiation and remodeling, resulting in secretion of CRTAC1 into the airway space rather than the circulation. Such loss of polar secretion could be due to disruption of positioning on T2AE cell‐specific microdomains on basal lamina (Sannes, 1984). In addition, CRTAC1 may interact with and deposit along with CFP at sites of alternative complement pathway activation. Such depletion would amplify the loss of production from T2AE cells and result in the profound drop in plasma CRTAC1 that correlates with the lesser drop in plasma CFP. The subset of patients recovering from COVID‐19 in whom CRTAC1 fails to return to normal may have continued disruption of T2AE cells or ongoing complement activation or both. We emphasize that we do not know the concentration of CFP in our long COVID samples and expect that the level is determined by whether increased rate of synthesis of CFP is enough to balance increased deposition.

Our study has limitations and leaves important questions open. First, our hospitalized COVID‐19 patients were enrolled at the beginning of the pandemic and may not be representative of severally ill COVID‐19 patients today who are infected with SARS‐CoV2 variants; treated by more effective strategies, including dexamethasone; and may have preexisting immunity. Second, although CRTAC1 concentration distinguished hospitalized patients with respiratory distress due to COVID‐19 from those with respiratory distress caused by other conditions, the non‐COVID‐19 patients were fewer in number and heterogenous. Some non‐COVID‐19 patients had low CRTAC1 concentrations, and some correlations between CRTAC1 concentration and measures of disease severity trended toward significance for the non‐COVID‐19 population as a whole. Additional studies are needed with enough patients with causes of respiratory distress other than COVID‐19 to determine the range of CRTAC1 concentrations in the other conditions, especially influenza and other viral pneumonias. Third, our long COVID patients were self‐referred and may not be representative of all patients recovering from COVID‐19. We found no correlations between CRTAC1 concentrations and the domains of the SF‐36 health survey. Determination of the reasons behind and significance of decreased CRTAC1 concentration in a subset of post‐COVID‐19 patients will require more detailed analysis of the roles of age, nature, and timing of the episode of COVID‐19, and conditions that drive plasma CRTAC1 concentration up, such as osteoarthritis (Styrkarsdottir et al., 2021, 2023; Szilagyi et al., 2023; Tardif et al., 2022), or down, such idiopathic pulmonary fibrosis (Mayr et al., 2021) or COPD (this study). Importantly, longitudinal studies of CRTAC1 concentrations are needed post‐COVID‐19 in patients with and without symptoms of long COVID. One possible outcome is that CRTAC1 concentrations recover more slowly in a subset of long COVID patients than those without long COVID. If so, does the concentration ultimately level out and at what level? Because genetic variability of components of the alternative complement pathway is common, with up to 8% of the population harboring a variant with the potential to be associated with a complement‐associated disease (Rodriguez de Cordoba, 2023), analysis of complement activation, and genomic studies should be considered in patients with persistently low CRTAC1. Finally, the contributions of low plasma CRTAC1 to the pathophysiology of COVID‐19 and the aftermath are not known. Although CRTAC1 knockout mice have been reported not to have lung abnormalities (Sato et al., 2011), the lungs of such mice have not been challenged. Studies of models of COVID‐19 and other respiratory conditions in knockout animals are needed to learn if and how absence of CRTAC1 contributes to disease severity.

## AUTHOR CONTRIBUTIONS

Conceptualization/design: MWJ, DFM; data acquisition/curation: MWJ, JB, LKM, KAO, RV, AT, ARA; formal analysis/interpretation: MWJ, JB, YVB, MSS, PJF, BSS, JCC, RS, AJ, DFM; funding acquisition: AJ, JCC, BSS; investigation: MWJ, JB, LKM, YVB, RV, AT, ARA, PJF, DFM; methodology: MWJ, DFM; project administration: DFM, AJ, BSS; resources: AJ, JCC; supervision: DFM, AJ, RS, JCC, BSS; validation: MWJ, JB, LKM; visualization: MWJ, YVB, MSS; writing (original draft): MWJ, DFM; writing (review/editing/revision): JB, YVB, AJ, PJF, KAO, BSS, RS, JCC, LKM, MSS, RV, AT, ARA; approval of final version and agreement to be accountable: MWJ, JB, LKM, YVB, MSS, KAO, RV, AT, ARA, PJF, BSS, JJC, RS, AJ, DFM.

## FUNDING INFORMATION

This work was funded by the National Institutes of Health R01‐AI173035 (AJ), K01‐HL130704 (AJ), R01‐HL160661 (AJ), and P41‐GM108538 (JJC), and the Morgridge Institute for Research.

## Supporting information


Data S1.
