# Supplementary figures and images for: Decreased plasma cartilage acidic protein 1 in COVID‐19
Source: Physiol Rep. 2023 Sep 4;11(17):e15814. doi: 10.14814/phy2.15814 (PMC10477339; doi:10.14814/phy2.15814)

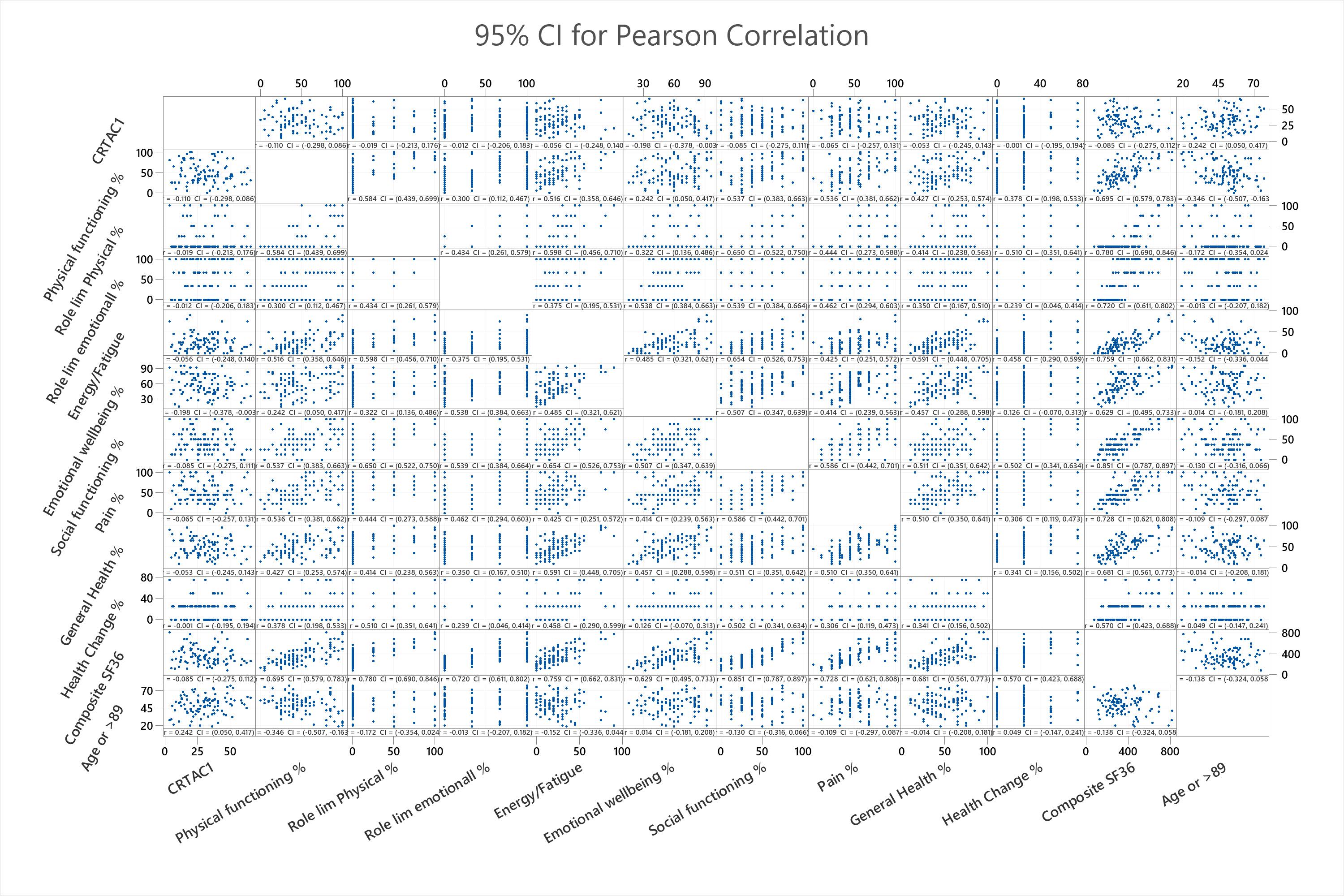

Supplement: Supplementary file 1 — Data S1. [file PHY2-11-e15814-s001.zip › phy215814-sup-0004-Supinfo4.jpg]
